# Post-Exercise Milk Consumption Behavior and Health- and Performance-Related Perceptions Among Collegiate Baseball Players: An Exploratory Cross-Sectional Study

**DOI:** 10.3390/nu18213609

**Published:** 2026-10-30

**Authors:** Tsukasa Yamamoto, Manato Seguchi, Yoko Iio, Mamoru Tanaka, Hana Kozai, Harumi Ejiri, Yuka Aoyama, Yukihiro Mori, Morihiro Ito

**Affiliations:** 1 Center for Nursing Practicum Support, Chubu University, 1200 Matsumoto-cho, Kasugai-shi 487-8501, Aichi, Japan; 2 Graduate School of Life and Health Sciences, Chubu University, 1200 Matsumoto-cho, Kasugai-shi 487-8501, Aichi, Japan; 3 Support for Pioneering Research Initiated by the Next Generation (SPRING), Chubu University, 1200 Matsumoto-cho, Kasugai-shi 487-8501, Aichi, Japan; 4 Department of Lifelong Sports and Health Sciences, College of Life and Health Sciences, Chubu University, 1200 Matsumoto-cho, Kasugai-shi 487-8501, Aichi, Japan; 5 Department of Food and Nutritional Sciences, College of Bioscience and Biotechnology, Chubu University, 1200 Matsumoto-cho, Kasugai-shi 487-8501, Aichi, Japan; 6 Department of Nursing, College of Life and Health Sciences, Chubu University, 1200 Matsumoto-cho, Kasugai-shi 487-8501, Aichi, Japan; 7 Department of Clinical Engineering, College of Life and Health Sciences, Chubu University, 1200 Matsumoto-cho, Kasugai-shi 487-8501, Aichi, Japan; 8 Department of Biomedical Sciences, College of Life and Health Sciences, Chubu University, 1200 Matsumoto-cho, Kasugai-shi 487-8501, Aichi, Japan

**Keywords:** collegiate athletes, milk consumption, nutrition behavior, health perception, performance perception, sports nutrition, health promotion, dietary behavior

## Abstract

Background/Objectives: The relationship between nutrition-related behavior and perceptions of specific foods is unclear. We examined whether distributions of milk-related perceptions regarding heat-illness prevention and exercise performance differed according to habitual post-exercise milk consumption status. Methods: In this exploratory cross-sectional study of 49 male collegiate baseball players, participants who answered “yes” to both post-exercise milk-consumption items were classified into the Milk group (*n* = 24), whereas those who answered “no” to both were classified into the Non-milk group (*n* = 25). Primary outcomes were milk-related perceptions of heat-illness prevention and exercise performance (Negative/Neutral/Positive). Between-group response distributions were assessed, with magnitude of association quantified; sensitivity analyses used Firth-penalized logistic regression (Positive vs. Neutral/Negative). Results: For heat-illness-prevention-related perception, responses in the Milk group were Positive in 54.2%, Neutral in 45.8%, and Negative in 0.0%, compared with 12.0%, 72.0%, and 16.0%, respectively, in the Non-milk group. For performance-related perception, corresponding Positive responses were 37.5% and 8.0%, respectively. Sensitivity analyses showed associations in the same direction. Conclusions: Habitual post-exercise milk consumption was associated with a higher frequency of Positive milk-related perceptions and different overall response distributions. These findings describe a behavioral–perceptual association and do not establish that milk consumption itself improves perceptions, prevents heat illness, or enhances exercise performance.

## 1. Introduction

Appropriate nutrition is important for athletes not only to maintain and improve competitive performance but also to support post-exercise recovery, reduce the risk of injury and illness, and promote long-term health [1,2]. However, evidence-based nutrition recommendations are not always translated into practice in real-world sports settings. Systematic reviews of athletes’ nutrition knowledge have shown that sports nutrition knowledge is often suboptimal and that its association with actual dietary behavior is not consistently strong [3,4]. Recent reviews have further emphasized that dietary behavior among team-sport athletes is shaped by multiple factors, including the sporting environment, time constraints, information from others, and individual beliefs and expectations [5].

These observations suggest that supporting athletes’ health requires attention not only to what athletes consume but also to how they perceive foods and nutrition-related behaviors. Studies of collegiate athletes have shown that food choices are influenced by expected health and performance benefits, convenience, and recommendations from others, and that dietary behavior and food-related perceptions are closely intertwined [6]. In collegiate baseball players, attitudes toward healthy eating, subjective norms, and perceived behavioral control have also been associated with intentions to adopt healthy dietary behaviors [7]. Together, these findings suggest that athletes’ nutrition-related behaviors and perceptions may be interrelated rather than linked by a simple unidirectional relationship.

Nutrition education and counseling can improve athletes’ nutrition knowledge, but greater knowledge does not necessarily translate directly into behavior change [8,9]. Systematic reviews of sports nutrition education have also highlighted limitations in the validity of assessment instruments and uncertainty regarding how changes in knowledge translate into changes in behavior [8]. Accordingly, the design of future nutrition-education and health-promotion interventions should be informed by an understanding of how nutrition-related behaviors and health-related perceptions are related in real-world sports settings.

Milk is one beverage commonly consumed after exercise. It contains water, carbohydrates, high-quality protein, sodium, potassium, and other nutrients and has been studied extensively as a post-exercise recovery beverage [10]. Following exercise-induced dehydration, low-fat milk has been associated with lower urine output than water or sports drinks, suggesting greater fluid retention [11]. In a randomized trial using the Beverage Hydration Index, milk also showed greater fluid-retention properties than water [12]. Post-exercise milk consumption has additionally been reviewed in relation to rehydration, muscle protein synthesis, glycogen resynthesis, and recovery of muscle function [10,13]. These studies, however, address the objective physiological properties of milk and its potential role in recovery, which is distinct from the question of how athletes perceive milk in relation to health and performance.

This distinction is particularly relevant for athletes who train and compete in hot environments. During prolonged outdoor activity in hot and humid conditions, appropriate hydration and heat-management strategies are important for health protection. The present study did not evaluate whether milk consumption prevents heat illness. Instead, it examined whether perceptions of milk—particularly perceptions related to heat-illness prevention and exercise performance—differed between athletes who habitually consumed milk after exercise and those who did not. Characterizing these perception patterns may provide useful baseline information for the development of evidence-based sports nutrition education.

Although athletes’ nutrition knowledge, food choices, dietary behaviors, and health-related beliefs have been widely studied [3,4,5,6,7,8,9], few studies have examined how a specific habitual post-exercise dietary behavior coexists with food-specific health- and performance-related perceptions. This distinction is relevant because nutrition education may be more effective when existing behaviors and perceptions are understood together rather than assuming that knowledge alone determines dietary behavior. In addition, retaining the original Negative, Neutral, and Positive response categories may reveal distributional patterns that would be obscured by simple binary classification. Most previous research on milk has focused on objective physiological outcomes such as rehydration and recovery [10,11,12,13], leaving the relationship between habitual consumption behavior and athletes’ own perceptions insufficiently characterized.

Therefore, this exploratory study aimed to characterize the distributions of milk-related heat-illness-prevention and performance perceptions according to habitual post-exercise milk consumption status among male collegiate baseball players. The contribution of the present study lies not in establishing a physiological benefit of milk or a causal effect of milk consumption on perceptions, but in characterizing how a habitual post-exercise nutrition behavior and milk-related perceptions are distributed together in a real-world collegiate sport setting. The original three response categories—Negative, Neutral, and Positive—were retained for both primary outcomes. Because of the cross-sectional design, the study was not intended to determine whether milk consumption shaped perceptions or whether pre-existing perceptions influenced milk-consumption behavior.

## 2. Materials and Methods

### 2.1. Study Design and Participants

This exploratory cross-sectional study included male collegiate baseball players from a single university in Aichi Prefecture, Japan. Participants were recruited from one collegiate baseball team that was accessible to the research group during the study period; recruitment was therefore team-based rather than designed to represent collegiate baseball players nationally or across different competitive levels. Sixty-two participants were enrolled. After exclusion of participants with incomplete questionnaire responses or missing required measurement data, 50 participants remained. One additional participant was excluded because records from the body-composition assessment indicated that the reliability of the measurement could not be ensured. The final analytic sample therefore comprised 49 participants. Self-reported milk intolerance or milk allergy was an exclusion criterion; however, no participant in the final analytic sample met this criterion. Because the participating baseball team consisted exclusively of male players, all study participants were male. Because this was an exploratory study conducted within a single collegiate baseball team, the final analytic sample was determined by the number of eligible and available players during the study period. No formal sample-size target was used to determine recruitment for the present study.

The study was conducted in accordance with the Declaration of Helsinki and was approved by the Institutional Review Board of Chubu University (approval numbers 20220001 and 20230056). Before participation, the study purpose and procedures, the voluntary nature of participation, the right to withdraw, and the protection of personal information were explained both orally and in writing. Written informed consent was obtained from all participants.

### 2.2. Data Collection

Data were collected in September 2024. A self-administered questionnaire was delivered using Google Forms and collected information on participant characteristics, habitual post-exercise milk consumption, milk-related perceptions, intention to continue milk consumption, and selected nutrition-, hydration-, and heat-health-related characteristics. The post-exercise milk-consumption items referred to habitual behavior after exercise in general and were not restricted to a specific practice session or game. Some heat-related background questions referred to practice and/or competition contexts, whereas the primary heat-illness-prevention perception item assessed a general perception of milk and was not tied to a specific practice or game.

Body composition was assessed before club activities using a body-composition analyzer (innerScan DUAL, RD-803L; TANITA Corporation, Tokyo, Japan). Body weight and muscle mass were recorded. Percentage body fat was not included in the study dataset and therefore could not be reported or analyzed. Height was not measured as part of the body-composition assessment protocol and was obtained from the self-administered questionnaire. BMI was therefore calculated as body weight (kg)/height (m)^2^ using self-reported height and measured body weight at baseline. Before measurement, participants received standardized instructions and completed the assessment according to the specified procedure; measurement values were then submitted via a dedicated application. The questionnaire used in this study is provided in Supplementary Material File S1.

### 2.3. Classification of Habitual Post-Exercise Milk Consumption

Habitual post-exercise milk consumption status was used as the primary grouping variable. The questionnaire included two items: “Do you consume ≥200 mL of milk immediately after exercise?” and “Do you have a habit of drinking milk immediately after exercise on ≥5 days per week?” Participants who answered “yes” to both items were classified into the Milk group. The 200-mL threshold was selected as a pragmatic operational criterion approximating a typical single-serving volume of milk in Japan; it was not intended to represent a physiological efficacy threshold. The ≥5-days-per-week threshold was used as an operational definition to distinguish regular consumption from occasional consumption. Participants who answered “no” to both items were classified into the Non-milk group. The final sample comprised 24 participants in the Milk group and 25 in the Non-milk group.

Milk intake volume and frequency were self-reported. Detailed information on milk type, fat content, fortification, brand, or nutrient composition was not collected because the original questionnaire focused on the presence and regularity of post-exercise milk consumption rather than on the nutritional composition of the consumed product. Consequently, differences in nutrient composition by milk type could not be evaluated. No dietary or exercise intervention was implemented, and no restrictions were placed on participants’ usual training or daily activities.

The two milk-consumption items were presented as separate questions to participants (Supplementary Materials) and were jointly used to define the primary Milk/Non-milk grouping variable. No alternative auxiliary milk-consumption variable was used to redefine group membership.

### 2.4. Assessment of Milk-Related Perceptions

The primary focus of this study was the distribution of milk-related health and performance perceptions according to habitual post-exercise milk consumption status. The questionnaire included three study-specific single-item questions: two primary outcome items and one secondary descriptive outcome item. Each item used three response categories—Negative, Neutral, and Positive—and the original three-category format was retained in the primary analyses. No formal psychometric validation, reliability assessment, or pilot testing was conducted for these items. Accordingly, they were interpreted as exploratory indicators of participants’ perceptions rather than as validated scales.

#### 2.4.1. Assessment of Heat-Illness-Prevention-Related Perception

Participants were asked whether they believed that milk was effective in preventing heat illness. Responses were categorized as Negative, Neutral, or Positive. This item was designated as the first primary outcome. It assessed participants’ perceived effectiveness of milk for heat-illness prevention and did not assess actual heat-illness prevention, hydration status, core body temperature, heat exposure, or heat-illness occurrence.

#### 2.4.2. Assessment of Performance-Related Perception

Participants were asked whether they believed that drinking milk improved exercise performance. Responses were categorized as Negative, Neutral, or Positive. This item was designated as the second primary outcome. It assessed participants’ perceived performance-related effects of milk and did not measure objective physical capacity or competitive performance.

#### 2.4.3. Assessment of Intention to Continue Post-Exercise Milk Consumption

Participants were asked whether they intended to continue drinking milk after exercise in the future. Responses were categorized as Negative, Neutral, or Positive. Because continuation intention conceptually overlaps with current habitual milk-consumption behavior, this item was treated as a secondary descriptive outcome rather than a primary outcome and was not included as a main target of multivariable analysis.

### 2.5. Participant Characteristics and Health-Behavior-Related Variables

Participant characteristics included age, BMI, muscle mass, and academic year. Selected nutrition- and heat-health-related variables obtained from the questionnaire were also summarized: self-perceived nutrition knowledge, heat-illness education, previous heat illness, and conscious post-exercise hydration behavior.

Nutrition knowledge was classified as Lower or Higher according to the analytic coding described in the Supplementary Materials; one participant had missing nutrition-knowledge data. Previous heat illness was classified as Yes or No; two participants did not respond to this item and were treated as having missing data. Conscious post-exercise hydration behavior was retained in its original three categories (No, Neutral, and Yes) for descriptive purposes. These background variables were used primarily to characterize the study population, and covariates were not selected on the basis of univariate *p*-values.

### 2.6. Statistical Analysis

Statistical analyses were performed using R version 4.6.0 within EZR version 1.70. Firth-penalized logistic regression was performed using the logistf package (version 1.26.1). The final analytic sample comprised 49 male collegiate baseball players, including 24 in the Milk group and 25 in the Non-milk group. Continuous variables are presented as mean ± standard deviation, and categorical variables as number and percentage. Given the limited sample size and exploratory design, the analyses were intended primarily to characterize response distributions and estimate exploratory associations rather than to provide precise population-level effect estimates.

The two primary outcomes were milk-related heat-illness-prevention perception and milk-related performance perception. For the primary analysis, each outcome retained its original three-category response structure (Negative, Neutral, and Positive), and a 2 × 3 contingency table was constructed according to habitual post-exercise milk consumption status. Because of the small sample size and sparse cells, between-group differences in response distributions were evaluated using the Fisher–Freeman–Halton exact test for 2 × 3 contingency tables. Cramér’s V was calculated as a measure of association.

The Milk/Non-milk grouping defined in Section 2.3 was used as the primary grouping variable. The two items used to define this grouping were not analyzed separately as auxiliary variables, and no additional 2 × 4 exact test was performed.

Because both primary outcomes were ordinal, ordinal logistic regression was considered as an exploratory secondary analysis. However, diagnostic evaluation raised concerns regarding model validity and stability. For heat-illness-prevention perception, the absence of Negative responses in the Milk group resulted in sparse data and separation-related instability at one cumulative threshold. For performance-related perception, threshold-specific coefficients differed substantially, raising concern about the proportional-odds assumption. Given these findings and the small sample size, ordinal logistic regression was not retained for the final analysis.

As a sensitivity analysis, each primary outcome was dichotomized as Positive versus Neutral/Negative, and Firth-penalized logistic regression was used to reduce small-sample bias and mitigate the effects of sparse cells and potential separation. For heat-illness-prevention perception, both a crude model including milk-consumption status alone and a minimally adjusted model including milk-consumption status, age, and heat-illness education were reported. Age and heat-illness education were selected on conceptual grounds rather than on the basis of univariate *p*-values, and the adjusted model included all 49 participants. For performance-related perception, a candidate adjusted model including age and nutrition knowledge was also examined; however, because the estimates were substantially imprecise and unstable in this small sample, only the crude Firth model was retained in the final sensitivity analysis. Firth model results are reported as odds ratios (ORs) with 95% profile penalized-likelihood confidence intervals (CIs). These sensitivity analyses were used to assess the consistency of the findings and were not intended to replace the primary three-category distributional analysis.

Continuation intention was treated as a secondary descriptive outcome because of its conceptual overlap with current habitual milk-consumption behavior. Its three-category distribution was summarized by Milk/Non-milk status and evaluated using an exact test and Cramér’s V. Missing data were not imputed. Analyses involving variables with missing values used available-case or complete-case data, as appropriate, and the corresponding analytic sample size was reported.

Because this was an exploratory study with two primary outcomes, no formal adjustment for multiple comparisons was applied. Interpretation emphasized response distributions, effect sizes, confidence intervals, and consistency across analyses rather than *p*-values alone. All findings were interpreted as associations or distributional patterns consistent with the cross-sectional design; no causal interpretation was made regarding whether milk consumption influenced perceptions or pre-existing perceptions influenced milk-consumption behavior.

## 3. Results

### 3.1. Participant Characteristics

The final analytic sample comprised 49 participants: 24 in the Milk group and 25 in the Non-milk group. Overall, the mean age was 20.08 ± 1.26 years, BMI 24.73 ± 2.22 kg/m^2^, and muscle mass 59.61 ± 4.66 kg. Mean age was 20.25 ± 1.22 years in the Milk group and 19.92 ± 1.29 years in the Non-milk group; mean BMI was 24.32 ± 2.03 and 25.12 ± 2.37 kg/m^2^, respectively; and mean muscle mass was 58.84 ± 4.88 and 60.34 ± 4.42 kg, respectively. Participants in both groups were distributed across all four academic years (Table 1).

Self-perceived nutrition knowledge was classified as Higher in 21/48 participants overall (43.8%), including 10/24 (41.7%) in the Milk group and 11/24 (45.8%) in the Non-milk group. Heat-illness education had been received by 33/49 participants (67.3%): 17/24 (70.8%) in the Milk group and 16/25 (64.0%) in the Non-milk group. Previous heat illness was reported by 30/47 participants (63.8%), including 13/23 (56.5%) in the Milk group and 17/24 (70.8%) in the Non-milk group. Conscious post-exercise hydration was reported by 30/49 participants (61.2%), including 16/24 (66.7%) in the Milk group and 14/25 (56.0%) in the Non-milk group (Table 1).

### 3.2. Heat-Illness-Prevention-Related Perception

**Figure 1:**
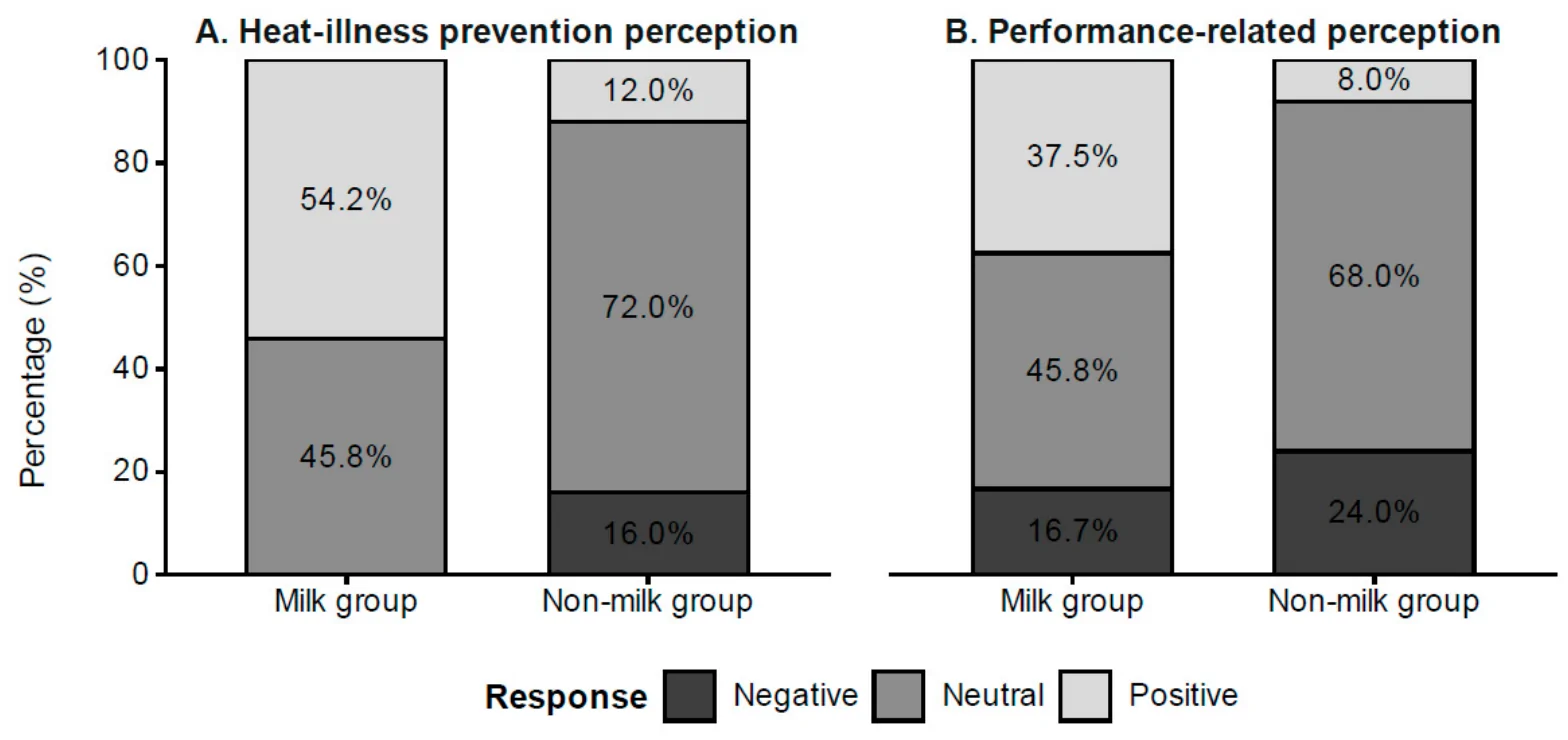
Distribution of milk-related heat-illness-prevention and performance perceptions according to habitual post-exercise milk consumption status. (**A**) Heat-illness-prevention perception; (**B**) performance-related perception. The Milk group (*n* = 24) comprised participants who answered “yes” to both “Do you consume ≥ 200 mL of milk immediately after exercise?” and “Do you have a habit of drinking milk immediately after exercise on ≥5 days per week?” The Non-milk group (*n* = 25) comprised participants who answered “no” to both items. Each bar shows the proportions of Negative, Neutral, and Positive responses within each group. Between-group differences in response distributions were evaluated using the Fisher–Freeman–Halton exact test. For heat-illness-prevention perception, *p* = 0.0015 and Cramér’s V = 0.493; for performance-related perception, *p* = 0.0449 and Cramér’s V = 0.353.

For heat-illness-prevention perception, responses in the Milk group were Negative in 0 participants (0.0%), Neutral in 11 (45.8%), and Positive in 13 (54.2%). Corresponding values in the Non-milk group were 4 (16.0%), 18 (72.0%), and 3 (12.0%), respectively (Table 2, Figure 1A). The three-category response distributions differed between groups (Fisher–Freeman–Halton exact test, *p* = 0.0015; Cramér’s V = 0.493).

### 3.3. Performance-Related Perception

For performance-related perception, responses in the Milk group were Negative in 4 participants (16.7%), Neutral in 11 (45.8%), and Positive in 9 (37.5%). Corresponding values in the Non-milk group were 6 (24.0%), 17 (68.0%), and 2 (8.0%), respectively (Table 2, Figure 1B). The Fisher–Freeman–Halton exact test yielded *p* = 0.0449, with Cramér’s V = 0.353.

### 3.4. Sensitivity Analyses

To assess consistency with the distributional differences observed in the primary analysis, each primary outcome was dichotomized as Positive versus Neutral/Negative and analyzed using Firth-penalized logistic regression (Supplementary Table S1). For heat-illness-prevention perception, the crude OR for a Positive response in the Milk group versus the Non-milk group was 7.55 (95% CI: 2.07–34.30, *p* = 0.0017). In the minimally adjusted model including age and heat-illness education, the OR was 8.11 (95% CI: 2.18–39.08, *p* = 0.0013), indicating an association in the same direction as the crude model.

For performance-related perception, the crude OR for a Positive response in the Milk group versus the Non-milk group was 5.76 (95% CI: 1.39–33.39, *p* = 0.0144). A candidate adjusted model including age and nutrition knowledge was also examined; however, because of substantial imprecision and instability in the estimates, only the crude model was retained in the final sensitivity analysis. These Firth regression analyses were used as supplementary sensitivity analyses to assess consistency with the primary three-category findings and were not intended to replace the primary analysis.

### 3.5. Intention to Continue Post-Exercise Milk Consumption

For intention to continue post-exercise milk consumption, responses in the Milk group were Negative in 4 participants (16.7%), Neutral in 5 (20.8%), and Positive in 15 (62.5%), whereas corresponding values in the Non-milk group were 4 (16.0%), 17 (68.0%), and 4 (16.0%), respectively (Supplementary Table S2). The between-group response distributions differed (exact test, *p* = 0.0011; Cramér’s V = 0.513). Because continuation intention conceptually overlaps with current habitual milk-consumption behavior, this variable was treated as a secondary descriptive outcome and was not considered a primary inferential target.

## 4. Discussion

This exploratory study examined the relationship between habitual post-exercise milk consumption and milk-related health and performance perceptions among male collegiate baseball players. The principal contribution of this study is the characterization of the coexistence of a habitual nutrition behavior and food-specific perceptions rather than demonstration of a causal or physiological effect of milk consumption. In the primary analysis, the original three response categories—Negative, Neutral, and Positive—were retained for both heat-illness-prevention and performance perceptions. Response distributions differed between the Milk and Non-milk groups for both primary outcomes. Cramér’s V was 0.493 for heat-illness-prevention perception and 0.353 for performance-related perception; Positive responses were more common in the Milk group, whereas Neutral responses were more common in the Non-milk group. These findings indicate that milk-related perceptions differ according to habitual post-exercise milk-consumption behavior. However, because of the cross-sectional design, the findings do not establish whether milk consumption shaped these perceptions or whether pre-existing perceptions influenced milk-consumption behavior.

A key feature of this study was the use of the original three-category response distributions rather than a primary binary comparison of Positive versus Neutral/Negative. In the Non-milk group, 72.0% of responses for heat-illness-prevention perception and 68.0% for performance-related perception were Neutral. Combining Neutral and Negative responses would have obscured this concentration of Neutral responses. Retaining all three categories was therefore appropriate for characterizing the distributional patterns central to the study question. However, the present data cannot determine whether Neutral responses reflected limited knowledge, uncertainty, low interest, withheld judgment, or other factors.

Athletes’ food choices are influenced by numerous factors beyond nutrition knowledge, including taste, convenience, cost, the sporting environment, social influences, and individual beliefs and expectations [14]. Birkenhead and Slater described athletes’ food choices as a multifactorial and dynamic process in which psychological, social, and economic factors interact with nutrition knowledge and beliefs [14]. Qualitative research in high-performance sport has similarly shown that adherence to nutrition guidelines is influenced by motivation, opportunity, time, social relationships, and sporting culture [15]. Accordingly, the differences in perception distributions observed between the Milk and Non-milk groups may be related to multiple background factors, including prior exposure to nutrition information, team practices, personal experiences and preferences, and advice from others.

Studies of collegiate athletes have also reported suboptimal sports nutrition knowledge and discrepancies between nutrition knowledge and dietary behavior [3,4,16]. Jagim et al. examined the relationship between sports nutrition knowledge and perceived energy and nutrient requirements among collegiate athletes and reported that athletes may not accurately perceive their own requirements [16]. Research on nutrition information sources and dietary habits among collegiate athletes has also shown that athletes obtain nutrition information from multiple non-expert sources and that scientific knowledge does not necessarily align with daily dietary behavior [17]. The present findings should therefore not be interpreted simply as reflecting differences in knowledge; rather, they may represent one aspect of health behavior involving how nutrition information, perceptions, and daily dietary practices are related.

Milk has primarily been studied from a physiological perspective as a post-exercise recovery beverage. Its potential role in rehydration and recovery has been reported [10,11,13] and Beverage Hydration Index studies have shown greater fluid retention with milk than with water [12]. These physiological findings, however, cannot be equated with the perception assessed in the present study—that milk is effective for preventing heat illness. We did not measure body temperature, sweat rate, blood- or urine-based hydration markers, heat-illness occurrence, or actual exercise performance. Accordingly, our findings do not demonstrate heat-illness-preventive or performance-enhancing effects of milk; they reflect participants’ responses to specific questions about the perceived health- and performance-related effects of milk. Distinguishing objective physiological effects from subjective perceptions is essential when interpreting these results.

Understanding these perceptions may nevertheless be relevant to health promotion and nutrition education in sports settings. Although nutrition-education interventions can improve athletes’ nutrition knowledge [8,9], increased knowledge does not necessarily lead directly to changes in dietary behavior. Dietary behavior is influenced by individual capability and motivation, food access, and the surrounding social environment [15]. Nutrition education should therefore consider not only the information provided but also athletes’ existing perceptions and the information sources that may shape them. The predominance of Neutral responses in the Non-milk group indicates, at minimum, a lower proportion of clearly positive perceptions than in the Milk group. Future research should investigate the knowledge, information sources, experiences, and preferences underlying these response patterns.

In the sensitivity analysis, each primary outcome was dichotomized as Positive versus Neutral/Negative and analyzed using Firth-penalized logistic regression. For heat-illness-prevention perception, the association with a Positive response was similar in direction in the crude model and the model minimally adjusted for age and heat-illness education. For performance-related perception, the crude model showed an association in the same direction. Although a candidate adjusted model including age and nutrition knowledge was examined for the performance outcome, the resulting estimates were too imprecise and unstable to retain. These Firth regression results should therefore be viewed as supplementary sensitivity analyses that are directionally consistent with the primary distributional findings. The wide 95% CIs further underscore that the central finding is the between-group pattern in the original three-category responses, not the magnitude of the ORs.

The distribution of intention to continue post-exercise milk consumption also differed between the Milk and Non-milk groups. However, current behavior and future intention are conceptually overlapping constructs: participants who already consume milk habitually may be more likely to report an intention to continue doing so. Continuation intention was therefore treated as a secondary descriptive outcome rather than a primary outcome, avoiding overinterpretation of this measure as a behavioral outcome independent of current habitual consumption.

This study has several strengths. First, it assessed habitual nutrition behavior and milk-related health and performance perceptions simultaneously in a real-world collegiate sports setting. Second, retaining the original Negative, Neutral, and Positive response categories avoided information loss from dichotomization and allowed the primary analysis to focus on the full response distribution. Third, the analytic strategy accounted for the small sample size and sparse cells by using exact tests and Cramér’s V for the primary analyses, with Firth-penalized logistic regression as a sensitivity analysis. Fourth, the study also collected contextual information on nutrition knowledge, heat-illness education, previous heat illness, and post-exercise hydration behavior, providing useful background for future research on factors related to athletes’ nutrition behaviors and perceptions.

This study also has important limitations. First, its cross-sectional design precludes determination of temporal order or causality between milk-consumption behavior and perceptions. Habitual milk consumption may have influenced perceptions, pre-existing perceptions may have influenced consumption behavior, or both may reflect common background factors such as exposure to nutrition information, team culture, or influence from coaches or family. Second, the sample was modest and restricted to 49 male collegiate baseball players from a single team in a limited geographic area. Because the study used an available team-based exploratory sample, the final sample size was constrained by the number of eligible players in the participating team rather than by a predetermined recruitment target. Consequently, the OR estimates from the Firth regression had wide 95% CIs, indicating limited precision, and robust adjustment for multiple potential confounders was not feasible. Generalizability to female athletes, other collegiate baseball teams, other sports, different playing positions, competitive levels, and geographic settings is therefore limited. Playing position was not recorded; because training and competition demands may differ by position, this unmeasured sport-specific factor may have contributed to heterogeneity in post-exercise behavior and perceptions. Third, the primary outcomes were study-specific single-item questions without formal validation, reliability assessment, or psychometric evaluation. They therefore capture responses to specific questions rather than the full constructs of health beliefs or performance-related beliefs. The validity and reliability of assessment instruments have also been identified as limitations in research on athletes’ nutrition knowledge [18]. Future studies should use multi-item or validated measures appropriate to the target population. Fourth, milk-consumption status and perceptions were self-reported and may be subject to recall and social-desirability bias. The Milk-group definition was operational, based on consuming ≥200 mL of milk immediately after exercise and doing so on ≥5 days per week; the 200-mL cutoff should not be interpreted as a biologically or physiologically validated threshold. Detailed milk type, fat content, fortification, exact timing, total intake, and co-consumed foods were not assessed. Consequently, the present findings cannot determine whether differences in milk composition contributed to consumption behavior or perceptions and should not be generalized to any specific milk formulation. Fifth, height was self-reported rather than measured using a standardized anthropometric procedure, which may have introduced measurement error into BMI, and detailed body-composition indices such as percentage body fat were not available for analysis. Sixth, several potentially relevant confounders were not measured or were not characterized in sufficient detail, including habitual dietary intake, total energy and protein intake, diet quality, training volume and intensity, use of other post-exercise or recovery beverages, prior formal nutrition education, and recommendations from coaches, dietitians, family members, or other information sources. These factors may influence both habitual milk consumption and perceptions of its potential benefits; therefore, substantial residual confounding cannot be excluded, and the observed associations should not be interpreted as independent effects of milk-consumption behavior. Finally, ambient temperature and humidity at individual practice sessions or games were not prospectively recorded. Because the present study assessed perceptions rather than actual heat exposure, physiological heat strain, or heat-illness occurrence, environmental conditions were not incorporated into the primary analyses.

Future studies should replicate these findings using validated measures in larger, multi-institutional and multi-sport samples. Longitudinal designs are needed to examine temporal changes in nutrition behavior and health- and performance-related perceptions. To distinguish perceptions from objective physiological effects, future research should also use prospective intervention designs, ideally randomized controlled trials, in which milk type and dose, timing of intake, exercise load, co-consumed foods, and environmental conditions are standardized or carefully measured. Appropriate comparator beverages and objective outcomes such as hydration markers, body-temperature responses, recovery indices, and sport-specific performance measures should be included. Combining such physiological outcomes with validated perception measures would help determine whether changes in beliefs correspond to measurable effects. Nutrition-education interventions should not aim simply to increase milk consumption; rather, they should support athletes in understanding the evidence-based roles of foods and beverages and evaluate how knowledge, perceptions, and behaviors change over time. From a health-communication perspective, it is particularly important to distinguish the physiological evidence regarding the role of milk in rehydration and post-exercise recovery [10,11,12,13] from the broader belief that milk prevents heat illness.

## 5. Conclusions

In this exploratory sample, habitual post-exercise milk consumers and non-consumers showed different distributions of milk-related perceptions regarding heat-illness prevention and exercise performance, with Positive responses more common in the Milk group and Neutral responses more common in the Non-milk group.

These observations characterize a behavioral–perceptual pattern within one male collegiate baseball team and should not be interpreted as evidence that milk consumption causes more favorable perceptions or produces physiological benefits. The cross-sectional design precludes causal inference regarding whether milk consumption influenced perceptions or perceptions influenced milk-consumption behavior, and the study did not assess actual heat-illness prevention, hydration status, body-temperature responses, or competitive performance.

Replication in larger and more diverse samples using validated measures and more detailed dietary, training, and environmental assessments is required. Sports nutrition education and health-promotion programs may benefit from considering athletes’ existing perceptions together with their dietary behaviors, in addition to providing evidence-based nutrition information.

## Figures and Tables

**Table 1:** Participant characteristics according to habitual post-exercise milk consumption status.

Characteristic	Overall (*n* = 49)	Milk Group (*n* = 24)	Non-Milk Group (*n* = 25)
Age, years	20.08 ± 1.26	20.25 ± 1.22	19.92 ± 1.29
BMI, kg/m^2^	24.73 ± 2.22	24.32 ± 2.03	25.12 ± 2.37
Muscle mass, kg	59.61 ± 4.66	58.84 ± 4.88	60.34 ± 4.42
Grade, *n* (%)			
1st year	14 (28.6)	6 (25.0)	8 (32.0)
2nd year	13 (26.5)	7 (29.2)	6 (24.0)
3rd year	12 (24.5)	5 (20.8)	7 (28.0)
4th year	10 (20.4)	6 (25.0)	4 (16.0)
Nutrition knowledge, *n* (%)			
Lower	27/48 (56.2)	14/24 (58.3)	13/24 (54.2)
Higher	21/48 (43.8)	10/24 (41.7)	11/24 (45.8)
Heat-illness education, *n* (%)			
No	16 (32.7)	7 (29.2)	9 (36.0)
Yes	33 (67.3)	17 (70.8)	16 (64.0)
Previous heat illness, *n* (%)			
No	17/47 (36.2)	10/23 (43.5)	7/24 (29.2)
Yes	30/47 (63.8)	13/23 (56.5)	17/24 (70.8)
Conscious post-exercise hydration, *n* (%)			
No	4 (8.2)	1 (4.2)	3 (12.0)
Neutral	15 (30.6)	7 (29.2)	8 (32.0)
Yes	30 (61.2)	16 (66.7)	14 (56.0)

Data are presented as mean ± standard deviation or *n* (%), as appropriate. BMI was calculated using self-reported height and baseline measured body weight. One participant in the Non-milk group had missing nutrition-knowledge data. Two participants did not respond to the previous heat-illness item and were treated as having missing data. Percentages are based on available cases.

**Table 2:** Distribution of primary milk-related perceptions according to habitual post-exercise milk consumption status.

Primary Outcome	Group	Negative, *n* (%)	Neutral, *n* (%)	Positive, *n* (%)	Exact *p*-Value	Cramér’s V
Heat-illness prevention perception	Milk group (*n* = 24)	0 (0.0)	11 (45.8)	13 (54.2)	0.0015	0.493
	Non-milk group (*n* = 25)	4 (16.0)	18 (72.0)	3 (12.0)		
Performance-related perception	Milk group (*n* = 24)	4 (16.7)	11 (45.8)	9 (37.5)	0.0449	0.353
	Non-milk group (*n* = 25)	6 (24.0)	17 (68.0)	2 (8.0)		

Percentages are calculated within habitual post-exercise milk consumption groups. Differences in the three-category response distributions were evaluated using the Fisher–Freeman–Halton exact test for 2 × 3 contingency tables. Cramér’s V was used as the measure of association. No primary outcome data were missing.

## Data Availability

The datasets generated and/or analyzed during the current study are not publicly available due to privacy and ethical restrictions but are available from the corresponding author on reasonable request.

## Supplementary Materials

The following supporting information can be downloaded at https://www.mdpi.com/article/10.3390/nu18213609/s1, File S1: Questionnaire sections containing variables analyzed or described in the present study; Table S1: Firth logistic regression sensitivity analyses for positive milk-related perceptions; Table S2: Distribution of intention to continue post-exercise milk consumption according to habitual post-exercise milk consumption status.

## Abbreviations

The following abbreviations are used in this manuscript:

BMI	Body mass index
CI	Confidence interval
OR	Odds ratio
SD	Standard deviation
