# Supplementary material for: Post-Exercise Milk Consumption Behavior and Health- and Performance-Related Perceptions Among Collegiate Baseball Players: An Exploratory Cross-Sectional Study
Source: Nutrients. 2026 Oct 30;18(21):3609. doi: 10.3390/nu18213609 (PMC13764907; doi:10.3390/nu18213609)
Supplement: Supplementary file 1 [file nutrients-18-03609-s001.zip › Supplementary_File_S1_v2.pdf]

Supplementary Questionnaire

本補足資料には、参加者に実際に提示した質問票のうち、本研究で解析または記述に使用した項目を含む関連セクションを、原質問票の順序および回答形式を保持して掲載する。質問票は日本語で実施され、本資料では英訳を併記する。解析に直接関係しない別テーマの質問票セクションは掲載していない。

This supplementary material reproduces the questionnaire sections containing variables analyzed or described in the present study, while preserving the original order and response format within those sections. The questionnaire was administered in Japanese, and English translations are provided alongside. Sections unrelated to the present study are not reproduced.

A. Basic participant characteristics

Original Japanese item and response options	English translation and response options
年齢<br><br>回答肢：自由記述／数値入力	Age<br><br>Response options: Open numeric response
学年<br><br>回答肢：1 年生 / 2 年生 / 3 年生 / 4 年生	Academic year<br><br>Response options: First year / Second year / Third year / Fourth year
学部<br><br>回答肢：経営情報学部 / 現代教育学部 / 工学部 / 国際関係学部 / 人文学部 / 生命健康科学部	Faculty / school<br><br>Response options: Management and Information Science / Contemporary Education / Engineering / International Relations / Humanities / Life and Health Sciences
居住形態<br><br>回答肢：その他 / 同居	Living arrangement<br><br>Response options: Other / Living with others

B. Nutrition-related attitudes and knowledge

Original Japanese item and response options	English translation and response options
食意識が高い方か<br><br>回答肢：思わない / あまり思わない / どちらでもない / 少し思う / そう思う	Do you consider yourself to have a high level of dietary awareness?<br><br>Response options: Do not think so / Do not really think so / Neither / Think so somewhat / Think so
朝食を取ることは重要だと思う<br><br>回答肢：当てはまらない / やや当てはまらない / どちらとも言えない / 少し当てはまる / 当てはまる	I think eating breakfast is important.<br><br>Response options: Does not apply / Applies somewhat poorly / Neither / Applies somewhat / Applies
昼食を取ることは重要だと思う<br><br>回答肢：当てはまらない / やや当てはまらない / どちらとも言えない / 少し当てはまる / 当てはまる	I think eating lunch is important.<br><br>Response options: Does not apply / Applies somewhat poorly / Neither / Applies somewhat / Applies
夕食を取ることは重要だと思う<br><br>回答肢：当てはまらない / やや当てはまらない / どちらとも言えない / 少し当てはまる / 当てはまる	I think eating dinner is important.<br><br>Response options: Does not apply / Applies somewhat poorly / Neither / Applies somewhat / Applies
栄養バランスは重要だと思う<br><br>回答肢：当てはまらない / やや当てはまらない / どちらとも言えない / 少し当てはまる / 当てはまる	I think nutritional balance is important.<br><br>Response options: Does not apply / Applies somewhat poorly / Neither / Applies somewhat / Applies
身体づくりにおいて食事の取り方は重要だと思う<br><br>回答肢：当てはまらない / やや当てはまらない / どちらとも言えない / 少し当てはまる / 当てはまる	I think dietary practices are important for body development.<br><br>Response options: Does not apply / Applies somewhat poorly / Neither / Applies somewhat / Applies
栄養について知識がある方だと思う<br><br>回答肢：当てはまらない / やや当てはまらない / どちらとも言えない / 少し当てはまる / 当てはまる	I think I am knowledgeable about nutrition.<br><br>Response options: Does not apply / Applies somewhat poorly / Neither / Applies somewhat / Applies
体力維持向上において食事の取り方は重要だと思う	I think dietary practices are important for maintaining and improving physical fitness.

Original Japanese item and response options	English translation and response options
回答肢：当てはまらない / やや当てはまらない / どちらとも言えない / 少し当てはまる / 当てはまる	Response options: Does not apply / Applies somewhat poorly / Neither / Applies somewhat / Applies
技術力向上のために食事の取り方は重要だと思う<br><br>回答肢：当てはまらない / やや当てはまらない / どちらとも言えない / 少し当てはまる / 当てはまる	I think dietary practices are important for improving athletic skills.<br><br>Response options: Does not apply / Applies somewhat poorly / Neither / Applies somewhat / Applies

C. Beverage intake, milk consumption, hydration, and milk-related perceptions

Original Japanese item and response options	English translation and response options
運動した 1 日 にどのくらい水を摂取しているか<br><br>回答肢：飲まない / 250 ml 未満 / 250 ml 以上 500 ml 未満 / 500 ml 以上 750 ml 未満 / 750 ml 以上 1000 ml 未満 / 1000 ml 以上	How much water do you consume in one day on a day when you exercise?<br><br>Response options: Do not drink / <250 mL / 250 to <500 mL / 500 to <750 mL / 750 to <1000 mL / ≥1000 mL
運動した 1 日 にどのくらいお茶を摂取しているか<br><br>回答肢：飲まない / 250 ml 未満 / 250 ml 以上 500 ml 未満 / 500 ml 以上 750 ml 未満 / 750 ml 以上 1000 ml 未満 / 1000 ml 以上	How much tea do you consume in one day on a day when you exercise?<br><br>Response options: Do not drink / <250 mL / 250 to <500 mL / 500 to <750 mL / 750 to <1000 mL / ≥1000 mL
運動した 1 日 にどのくらいスポドリを摂取しているか<br><br>回答肢：飲まない / 250 ml 未満 / 250 ml 以上 500 ml 未満 / 500 ml 以上 750 ml 未満 / 750 ml 以上 1000 ml 未満 / 1000 ml 以上	How much sports drink do you consume in one day on a day when you exercise?<br><br>Response options: Do not drink / <250 mL / 250 to <500 mL / 500 to <750 mL / 750 to <1000 mL / ≥1000 mL
運動した 1 日 にどのくらいエナジードリンクを摂取しているか<br><br>回答肢：飲まない / 250 ml 未満 / 250 ml 以上 500 ml 未満 / 500 ml 以上 750 ml 未満 / 750 ml 以上 1000 ml 未満 / 1000 ml 以上	How much energy drink do you consume in one day on a day when you exercise?<br><br>Response options: Do not drink / <250 mL / 250 to <500 mL / 500 to <750 mL / 750 to <1000 mL / ≥1000 mL
運動した 1 日 にどのくらい牛乳を摂取しているか<br><br>回答肢：飲まない / 250 ml 未満 / 250 ml 以上 500 ml 未満 / 500 ml 以上 750 ml 未満 / 750 ml 以上 1000 ml 未満 / 1000 ml 以上	How much milk do you consume in one day on a day when you exercise?<br><br>Response options: Do not drink / <250 mL / 250 to <500 mL / 500 to <750 mL / 750 to <1000 mL / ≥1000 mL
運動した 1 日 にどのくらい炭酸飲料を摂取しているか<br><br>回答肢：飲まない / 250 ml 未満 / 250 ml 以上 500 ml 未満 / 500 ml 以上 750 ml 未満 / 750 ml 以上 1000 ml 未満 / 1000 ml 以上	How much carbonated beverage do you consume in one day on a day when you exercise?<br><br>Response options: Do not drink / <250 mL / 250 to <500 mL / 500 to <750 mL / 750 to <1000 mL / ≥1000 mL
運動した 1 日 にどのくらいプロテインを摂取しているか<br><br>回答肢：飲まない / 250 ml 未満 / 250 ml 以上 500 ml 未満 / 500 ml 以上 750 ml 未満 / 750 ml 以上 1000 ml 未満 / 1000 ml 以上	How much protein drink do you consume in one day on a day when you exercise?<br><br>Response options: Do not drink / <250 mL / 250 to <500 mL / 500 to <750 mL / 750 to <1000 mL / ≥1000 mL
運動直後に 200 mL 以上の牛乳を摂取するか<br><br>回答肢：いいえ / はい	Do you consume at least 200 mL of milk immediately after exercise?<br><br>Response options: No / Yes
運動直後に牛乳を飲む習慣（週 5 日以上）はあるか<br><br>回答肢：いいえ / はい	Do you habitually drink milk immediately after exercise on at least 5 days per week?<br><br>Response options: No / Yes
運動直後、牛乳を飲むことに抵抗はあるか<br><br>回答肢：いいえ / どちらともいえない / はい	Do you have any reluctance to drink milk after exercise?<br><br>Response options: No / Neither / Yes
牛乳は熱中症予防に効果があると思うか<br><br>回答肢：いいえ / どちらともいえない / はい	Do you think milk is effective in preventing heat illness?<br><br>Response options: No / Neither / Yes
牛乳を飲むことで、運動パフォーマンスが向上すると考えるか<br><br>回答肢：いいえ / どちらともいえない / はい	Do you think drinking milk improves exercise performance?<br><br>Response options: No / Neither / Yes
運動後に意識して水分を摂取してるか<br><br>回答肢：いいえ / どちらともいえない / はい	Do you consciously consume fluids after exercise?<br><br>Response options: No / Neither / Yes

Original Japanese item and response options	English translation and response options
今後も運動後に牛乳を飲み続けていきたいか<br><br>回答肢：いいえ / どちらともいえない / はい	Would you like to continue drinking milk after exercise in the future?<br><br>Response options: No / Neither / Yes
牛乳を飲むことの有効性が出たら、今後も牛乳を飲み続けることへの思いは、どちらともいえない・いいえと回答した人は、「はい」へ変化するか はいと回答した人は、より強く飲み続けたと思うか<br><br>回答肢：いいえ / はい	If evidence of the effectiveness of drinking milk were demonstrated, would respondents who answered “neither” or “no” change to “yes,” and would respondents who answered “yes” feel more strongly about continuing to drink milk?<br><br>Response options: No / Yes

D. Heat-illness-related symptoms, education, and previous experience

Original Japanese item and response options	English translation and response options
部活動の練習や試合中に経験した自覚症状 [頭痛]<br><br>回答肢：よくある / ときどきある / ほとんどない / 全くない	Self-reported symptom experienced during club practice or games: Headache<br><br>Response options: Often / Sometimes / Rarely / Never
部活動の練習や試合中に経験した自覚症状 [口の中が乾く]<br><br>回答肢：よくある / ときどきある / ほとんどない / 全くない	Self-reported symptom experienced during club practice or games: Dry mouth<br><br>Response options: Often / Sometimes / Rarely / Never
部活動の練習や試合中に経験した自覚症状について [めまい]<br><br>回答肢：よくある / ときどきある / ほとんどない / 全くない	Self-reported symptom experienced during club practice or games: Dizziness<br><br>Response options: Often / Sometimes / Rarely / Never
部活動の練習や試合中に経験した自覚症状について [脱力感や倦怠感]<br><br>回答肢：よくある / ときどきある / ほとんどない / 全くない	Self-reported symptom experienced during club practice or games: Weakness or fatigue<br><br>Response options: Often / Sometimes / Rarely / Never
部活動の練習や試合中に経験した自覚症状について [集中力の低下や考えがまとまらない]<br><br>回答肢：よくある / ときどきある / ほとんどない / 全くない	Self-reported symptom experienced during club practice or games: Reduced concentration or difficulty organizing thoughts<br><br>Response options: Often / Sometimes / Rarely / Never
部活動の練習や試合中に経験した自覚症状について [吐き気]<br><br>回答肢：よくある / ときどきある / ほとんどない / 全くない	Self-reported symptom experienced during club practice or games: Nausea<br><br>Response options: Often / Sometimes / Rarely / Never
部活動の練習や試合中に経験した自覚症状について [筋肉のいれん]<br><br>回答肢：よくある / ときどきある / ほとんどない / 全くない	Self-reported symptom experienced during club practice or games: Muscle cramps<br><br>Response options: Often / Sometimes / Rarely / Never
部活動の練習や試合中に経験した自覚症状について [呼吸が以上になる]<br><br>回答肢：よくある / ときどきある / ほとんどない / 全くない	Self-reported symptom experienced during club practice or games: Abnormally difficult breathing<br><br>Response options: Often / Sometimes / Rarely / Never
部活動の練習や試合中に経験した自覚症状について [脈が速く、弱くなる]<br><br>回答肢：よくある / ときどきある / ほとんどない / 全くない	Self-reported symptom experienced during club practice or games: Rapid and weak pulse<br><br>Response options: Often / Sometimes / Rarely / Never
部活動の練習や試合中に経験した自覚症状について [唇のしびれ]<br><br>回答肢：よくある / ときどきある / ほとんどない / 全くない	Self-reported symptom experienced during club practice or games: Numbness of the lips<br><br>Response options: Often / Sometimes / Rarely / Never
部活動の練習や試合中に経験した自覚症状について [言動がおかしくなる]<br><br>回答肢：よくある / ときどきある / ほとんどない / 全くない	Self-reported symptom experienced during club practice or games: Abnormal speech or behavior<br><br>Response options: Often / Sometimes / Rarely / Never
部活動の練習や試合中に経験した自覚症状について [失神]<br><br>回答肢：よくある / ときどきある / ほとんどない / 全くない	Self-reported symptom experienced during club practice or games: Fainting<br><br>Response options: Often / Sometimes / Rarely / Never

Original Japanese item and response options	English translation and response options
部活動の練習や試合中に経験した自覚症状について [幻覚がみえる]<br><br>回答肢：よくある / ときどきある / ほとんどない / 全くない	Self-reported symptom experienced during club practice or games: Hallucinations<br><br>Response options: Often / Sometimes / Rarely / Never
[日本スポーツ協会の熱中症対策のマニュアル（HP や冊子）を見たことがある<br><br>回答肢：いいえ / はい	Have you seen the Japan Sport Association heat-illness prevention manual (website or booklet)?<br><br>Response options: No / Yes
熱中症対策の教育や指導経験の有無<br><br>回答肢：いいえ / はい	Have you received education or instruction on heat-illness prevention?<br><br>Response options: No / Yes
普段、猛暑時の警告やアドバイスの有無<br><br>回答肢：いいえ / はい	Do you usually receive warnings or advice during extremely hot weather?<br><br>Response options: No / Yes
部活動の練習または試合中に熱中症経験の有無<br><br>回答肢：いいえ / はい	Have you experienced heat illness during club practice or games?<br><br>Response options: No / Yes
熱中症経験数<br><br>回答肢：自由記述／数値入力	Number of heat-illness episodes<br><br>Response options: Open numeric response
部活動の練習や試合中に熱中症の診察の有無<br><br>回答肢：いいえ / はい	Have you received a medical examination for heat illness experienced during club practice or games?<br><br>Response options: No / Yes
熱中症の診察経験数<br><br>回答肢：自由記述／数値入力	Number of medical examinations for heat illness<br><br>Response options: Open numeric response

Supplementary Note. Analytic coding of questionnaire variables

Primary grouping variable: Participants were classified into the Milk group (n = 24) when they answered Yes to both participant-facing questionnaire items: (1) 「運動直後に 200 mL 以上の牛乳を摂取するか」 and (2) 「運動直後に牛乳を飲む習慣（週 5 日以上）はあるか」. Participants in the Non-milk group (n = 25) answered No to both items. Thus, the two questionnaire items jointly defined the primary Milk group/Non-milk group classification.

Post-exercise milk-consumption grouping items: The two binary grouping items were fully concordant in the final analytic dataset (Milk group: Yes/Yes, n = 24; Non-milk group: No/No, n = 25). They were used as the primary grouping definition and were not treated as a separate auxiliary four-level variable.

Primary outcomes: For analysis, responses to the two primary perception items were labeled Negative = No, Neutral = Neither, and Positive = Yes. The original three categories were retained in the primary analyses.

Nutrition knowledge: For the item 「栄養について知識がある方だと思う」, the first three response levels (当てはまらない / やや当てはまらない / どちらとも言えない) were analyzed as Lower and the last two levels (少し当てはまる / 当てはまる) as Higher. One participant had missing nutrition-knowledge data.

Previous heat illness: The formal questionnaire response options were いいえ / はい (No / Yes). Two participants did not answer this item and were treated as missing in the analysis. Analyses using this variable therefore used available cases.

Firth sensitivity analysis: For the sensitivity analysis, each primary perception outcome was dichotomized as Positive versus Neutral/Negative for Firth penalized logistic regression. This dichotomization was not used for the primary 2 × 3 analyses.
