# Supplementary material for: Post-Exercise Milk Consumption Behavior and Health- and Performance-Related Perceptions Among Collegiate Baseball Players: An Exploratory Cross-Sectional Study
Source: Nutrients. 2026 Oct 30;18(21):3609. doi: 10.3390/nu18213609 (PMC13764907; doi:10.3390/nu18213609)
Supplement: Supplementary file 1 [file nutrients-18-03609-s001.zip › Supplementary_tables_S1, S2_v2.pdf]

Supplementary Tables

Table S1. Firth logistic regression sensitivity analyses for positive milk-related perceptions

Outcome	Model	*n*	OR for Milk vs Non-milk	95% CI	*p*-value
Heat-illness prevention perception	Crude	49	7.55	2.07–34.30	0.0017
	Adjusted for age and heat-illness education	49	8.11	2.18–39.08	0.0013
Performance-related perception	Crude	49	5.76	1.39–33.39	0.0144

For the sensitivity analysis, each primary perception outcome was dichotomized as Positive versus Neutral/Negative for Firth-penalized logistic regression. This dichotomization was not used for the primary 2 × 3 analyses. Firth penalized logistic regression was used because of the small sample size and sparse outcome cells. The minimally adjusted model for heat-illness prevention perception included age and heat-illness education. CIs are profile penalized-likelihood confidence intervals.

Table S2. Distribution of intention to continue post-exercise milk consumption according to habitual post-exercise milk consumption status

Group	Negative, *n* (%)	Neutral, *n* (%)	Positive, *n* (%)	Exact *p*-value	Cramér's V
Milk group (*n* = 24)	4 (16.7)	5 (20.8)	15 (62.5)	0.0011	0.513
Non-milk group (*n* = 25)	4 (16.0)	17 (68.0)	4 (16.0)		

Percentages are calculated within habitual post-exercise milk consumption groups. Because continuation intention conceptually overlaps with current milk consumption behavior, this outcome was treated as secondary/descriptive. Differences in the three-category response distribution were evaluated using the Fisher–Freeman–Halton exact test. Cramér's V is reported as an effect-size measure.
